# Intra and interrater reliability and clinical feasibility of a simple measure of cervical movement sense in patients with neck pain

**DOI:** 10.1186/s12891-018-2287-0

**Published:** 2018-10-05

**Authors:** Isabelle M Werner, Markus J Ernst, Julia Treleaven, Rebecca J Crawford

**Affiliations:** 1 Department of Physiotherapy, Kantonsspital Schaffhausen, Geissbergstrasse 81, 8208 Schaffhausen, Switzerland; 2 Institute of Physiotherapy, Zurich University of Applied Sciences, Technikumstrasse 71, 8401 Winterthur, Switzerland; 3 Division of Physiotherapy, SHRS, University of Queensland, Brisbane, Australia; 4 Institute of Health Sciences, Zurich University of Applied Sciences, Technikumstrasse 71, 8401 Winterthur, Switzerland; 5 Faculty of Health Sciences, Curtin University, Perth, Australia

**Keywords:** Neck pain, Movement sense testing, Reliability, Feasibility, Proprioception, Sensorimotor control

## Abstract

**Background:**

Pattern tracing tasks can be used to assess cervical spine movement sense (CMS). A simple clinical measure of CMS (tracing fixed figure-of-eight (F8) and zigzag (ZZ) patterns with a head mounted laser) has been proposed and assessed in asymptomatic subjects. It is important to determine if examiner ratings of the traces are reliable and feasible for clinical use in those with neck pain. We therefore examined the intra- and inter-rater reliability of rating video recordings of the CMS tasks, and the feasibility of undertaking the tests in clinic by comparing slow motion versus real-time video ratings.

**Methods:**

Cross-sectional study examining neck pain subjects from a physiotherapy clinic. F8 and ZZ patterns traced with a head-mounted laser pointer at two velocities (accurate; accurate & fast) were videoed and later examined. *Time* (total time taken to complete the pattern), *error frequency* (number of deviations) and *error magnitude* (sum of deviations multiplied by distance from the central line) were measured. Two assessors independently evaluated the laser tracing videos in slow motion; a third rated the videos in real time. Intraclass correlation coefficients (ICC) and standard error of measurements (SEM) were calculated for intra- and inter-tester reliability, and feasibility.

**Results:**

Twenty neck pain patient (13 women) videos were assessed. Intra-and inter-rater reliability was substantial to almost-perfect (ICC 0.76–1.00; SEM < 0.01–2.50). Feasibility was moderate to almost-perfect (ICC 0.54–1; SEM <  0.01–2.98).

**Conclusions:**

Video (slow motion) ratings of time and errors for F8 and ZZ movement patterns in neck pain subjects showed high intra and inter-rater reliability. Achieving reliable ratings in clinic (real-time) appears feasible. Synthesising our results, the most reliable and feasible CMS ratings appear to be when the subject uses accurate rather than accurate and fast execution. The ZZ movement pattern may be superior to F8 in terms of rating. *Time and error frequency* for tracing F8 and ZZ as accurately as possible in determining CMS appears promising for use in clinic. Future research directions were identified.

## Background

Neck pain is a common musculoskeletal disorder with a global prevalence of around 5 % (women 5.8%, men 4.0%) [1]. It is a disabling condition with one of the highest socioeconomic burdens globally and is forecast to escalate with the world’s ageing population [2]. Neck pain is categorised into: pain secondary to an identifiable pathology like cervical myelopathy, neoplastic conditions, upper cervical ligamentous instability, vertebral artery insufficiency or inflammatory/systemic disease [3]; and non-specific neck pain with a poorly understood causation and into which the majority of sufferers are categorised. There is a mounting need to better understand important factors influencing non-specific neck pain (referred to as neck pain to follow).

Neck pain is a multifactorial condition with some patients experiencing symptoms due, at least in part, to proprioceptive dysfunction [4, 5] that can manifest as poor cervical position and/or movement sense [6]. Highly dense muscle spindles particularly in the sub-occipital muscles provide essential proprioceptive input for sensorimotor control [6–9]. In association with vestibular and visual reception, cervical proprioception contributes to optimising head and neck control [6–11]. However, such neuromotor control mechanisms can be disrupted with trauma [5, 12, 13], morphological changes in neck muscles [5], pain [5, 12, 13], inflammation [12, 13], fatigue [5, 12, 13], and/or where pathophysiological changes of the peripheral or central nervous system exist [12]. Negative long-term consequences of impeded proprioception, such as susceptibility to further injury, recurrence, and chronicity, have been shown [12] and form an important factor in considerations for rehabilitation. Integrating treatments targeting postural stability [6], cervical position sense [6], movement sense [6], head-eye coordination (including gaze stability) [6] and movement control are recommended in managing neck pain conditions [9, 13–15].

Cervical movement sense is defined as the ability to smoothly and accurately move the head/neck to a given pattern [16]. To date, several different methods to assess cervical movement sense have been used but all use head-mounted motion sensors and dedicated software to track, measure and calculate head motion accuracy; these methods have all shown reduced movement accuracy in neck pain subjects [16–20]. The measurement most studied is called the “Fly” and is purported to be the best test to differentiate asymptomatic from neck pain subjects and further to distinguish between neck pain subgroups like whiplash associated disorder (WAD) and non-specific neck pain [16, 20]. However, these tests require equipment that is generally cost-prohibitive for clinical practise. Consequently, a cost-effective and simple alternative for clinical use, has been promoted by Pereira et al. [21] based on a preliminary study examining asymptomatic subjects. Given the tasks and methodology, to what the subject is asked to perform, is similar with respect to previous work [19, 22], the primary difference here is the method of analysis of that performance. Therefore, it is important to establish if clinicians are able to reliably assess CMS (considering pattern and task type) using this simplified method of analysis and to explore the feasibility of using these tests in real time in the clinic by assessing subjects with neck pain. Thus the aim of this study was to determine the inter- and intra-rater reliability while rating videos in slow motion, and their feasibility when rating the videos in real-time. The influence of pattern shape (F8 and ZZ) and task type (accurate or accurate & fast) were considered.

## Methods

This observational, cross-sectional study consecutively recruited consenting neck pain subjects (non-specific or whiplash associated disorder (WAD)) attending the physiotherapy department of the Schaffhausen, Canton Hospital, Switzerland from April to October 2017. The clinic receives patients on referral from medical doctors that are internal and external to the hospital. Additional advertisements to address employees of all hospital departments were e-mailed. The ethics committee of the Canton of Zurich approved the study, and all patients signed their informed consent prior to participation.

Included were adults of either gender, aged 18 years or older with a Neck Disability Index score [23–25] of at least five points (or 10%). Subjects had to be suffering from WAD II (according to Quebec task force [26]) or non-specific neck pain for at least 3 months, were not familiar with movement sense tracking and were able to read and communicate in German.

Excluded were subjects with specific neck pain conditions like fractures, osteoporosis, myelopathy, nerve root entrapment, or WAD III or higher; Disorders of the ear, nose or throat resulting in vertigo or dizziness, like sudden hearing loss, Meniere’s disease or Tinnitus; Systemic diseases associated with neck pain like diabetes and rheumatoid arthritis; Neurologic diseases like multiple sclerosis or stroke affecting cervical spine musculature; Manual treatment of the cervical spine within 3 days prior to the measurements; and medication with potential to affect perception like Naproxen or opioids (e.g. Tramadol).

### Testing procedure for video capture of CMS

**Fig. 1:**
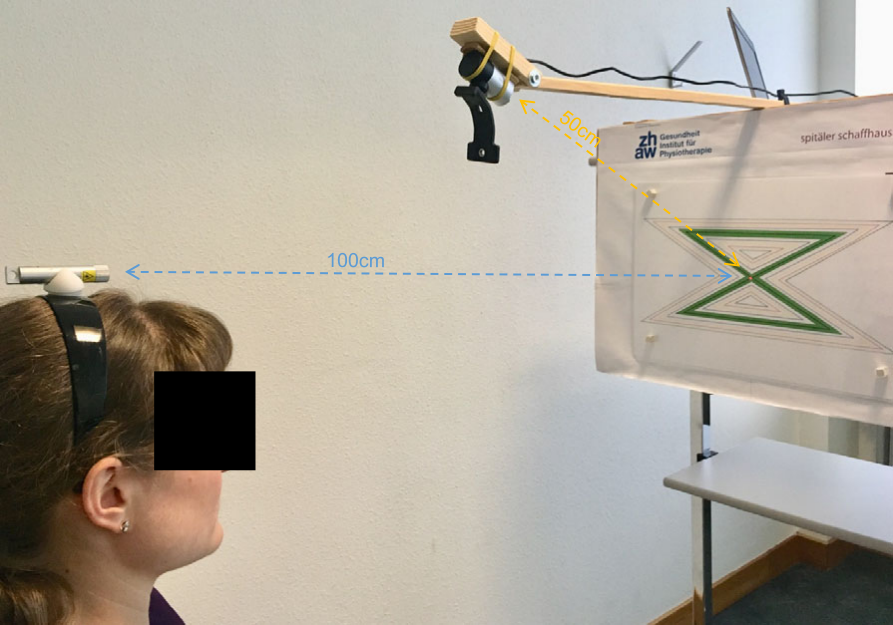
Test set-up. Subject sitting on a chair with LASER-Pointer on her head, at 100 cm distance from the ZZ-pattern. Laptop connected to a webcam at a distance of 50 cm from the centre of the pattern

Movement tests were undertaken in random order. The subject sat on a chair (with backrest) positioned 1 metre from a vertical wall to which the test patterns were fixed. Patterns were printed on A3 paper where a 5 mm thick black band (F8) and 10 mm thick green band (ZZ) represented the central (main) pattern. The F8 pattern was 13 cm high and 34.5 cm wide, with a total inner zone length of 94 cm. The ZZ pattern was 13 cm high and 23.4 cm wide with 23.4 cm long horizontal lines, 26.6 cm long diagonal lines and a total inner zone length of 100 cm. Both patterns had five thinner additional lines every 5 mm to both sides from the main line to distinguish five zones of deviation. With a laser pointer affixed to their forehead, subjects were instructed to follow the bands of each pattern: “as accurately as possible”, or “as accurately and fast as possible” and in two directions, clockwise or counter-clockwise to start from the centre of each pattern. Subjects were allowed to practise each task once. For all tests, the laser point tracing of the pattern was videoed using a webcam (Microsoft LifeCam Studio 1080p HD Sensor) positioned at 0,5 m in front of the patient (see Fig. 1). Video files were saved on a WINDOWS-Laptop. A pattern was considered completed when the subject returned to the central starting position.

### Evaluation of video capture of CMS tests by blinded raters

Video files were evaluated independently by two raters (R1 and R2) in slow motion at 1/8th of normal speed using the programme SMIPlayer (https://www.smplayer.info). All subjects were rated and results compared to determine inter-rater reliability. All videos from three randomly selected subjects were re-evaluated 4 weeks later by each rater blind to their initial results in order to determine intra-rater reliability. To reduce work-up bias, raters were blinded to other subject characteristics. Raters had received sufficient time for training to count error frequency by zone using twelve test videos. In determining feasibility, a third rater (R3; IMW) with similar pre-study training, determined time per subject at the time of recording in clinic and using the video replayed in real-time directly following the recording to determine error frequency.

### Outcome measures

Time, error frequency, and error magnitude while tracing the F8 and ZZ patterns were used to determine intra and interrater reliability and feasibility. *Time* was defined as tracing from the centre of the pattern once either into clockwise or counter-clockwise direction by stopping again at the centre of the pattern. *Error frequency* measured the number of errors occurring for each pattern tracing, defined by the laser pointer leaving/exceeding the pattern inner zone (F8 = 5 mm; ZZ = 10 mm). *Error magnitude* reflected by a composite error score, which comprises the sum of the product of error frequency times the zone (maximum of five), was additionally assessed. For example, number of errors occurring in zone 1 was multiplied by one, errors in the second zone by two, and so on. In addition, age, duration of pain and dizziness, current pain and dizziness (both separately using a visual analogue scale (VAS) [27]), traumatic/non-traumatic injury, which medication they were taking, NDI-G and the Dizziness Handicap Inventory – German version [28] (DHI-G) were recorded.

*Interpreting NDI-G and DHI-G:* While benchmarks for the NDI-G are not defined, recommendations interpret 0–4 points as no disability, 5–14 points as mild disability, 15–24 points as moderate disability, 25–34 points as severe disability, and 35–50 points as completely disabled [23, 24]. DHI-G is a reliable German version of the DHI used to assess the disability of patients suffering from dizziness [28]. Tesio et al. [29] developed a short form version of the English DHI where a score of 13 represents no disability and zero indicates being completely disabled secondary to dizziness. Without a validated German DHI-short form to use, the equivalent items used in the English short form were selected to represent a German DHI-short form.

### Data processing and analysis

Outcome variables were initially tested for any directional effects (clockwise/counter-clockwise) using paired Wilcoxon signed-rank tests. As no directional effects were found, results of both directions were combined for analyses.

Four variables were recorded for each of *time, error frequency* and *error magnitude*: two patterns (F8, ZZ) and two movement velocities (accurate, accurate & fast). The intraclass correlation coefficient (ICC) for agreement was used to determine intra- and inter-rater reliability. Both velocities (accurate and accurate &fast) were combined for intra-rater reliability, resulting in 12 observations (3 subjects × 2 ratings × 2 pattern) for each rater and outcome variable. Inter-rater reliability was based on 160 observations (20 subjects × 2 ratings × 2 patterns × 2 velocities) for each outcome variable. The standard error of measurement (SEM) as a measure of absolute reliability in the unit of the test was computed by using the formula: SD x square root of (1 –ICC) [30, 31]. ICC values obtained were interpreted to be moderate (between 0.4 and 0.59), substantial (0.6 and 0.79), and almost-perfect (0.8 or more) [31, 32].

To examine feasibility, real time ratings of time and error frequency were compared to final slow motion video ratings of each of the two video raters using the ICC agreement and the standard error of measurement (SEM) [30]. Error magnitude was not considered feasible to be achieved in real-time and was consequently omitted from this analysis of feasibility.

All analysis was conducted by using Cran-R version 3.4.1 [33] including the packages “psy” and “boot” [34, 35].

## Results

Twenty-seven subjects were recruited and 20 progressed after application of exclusion criteria where subjects with tinnitus (× 2), NDI-score < 5 points (× 2), and Diabetes type II (× 1), unable to communicate in German (× 1), and who were unwilling to participate (× 1) were excluded. Demographic data is shown in Table 1.

**Table 1:** Demographic and movement sense data of neck pain patients

Demographic data		*n* = 20		
		Mean	SD	Range
Age		40.1	12.35	38
Gender F/M		13/7	NA	NA
NDI/50		11.45	4.77	34
DHIsf/13		10.95	1.86	5
Duration pain (months)		70.13	63.49	202
Duration dizziness (months)		24.95	47.99	164
Traumatic/Idiopathic neck pain		12/8	NA	NA
Current pain (VAS)		33	20.9	80
Current dizziness (VAS)		0.23	0.54	1.5
Medication (Yes/No)		2/18	NA	NA
Time (sec)	F8 acc	28.88	11.28	44.45
	F8 acc-fast	11.40	6.22	21.06
	ZZ acc	24.85	12.68	54.48
	ZZ acc-fast	9.67	4.67	20.08
Error frequency	F8 acc	29.00	7.56	24.5
	F8 acc-fast	15.95	4.05	13.5
	ZZ acc	13.98	6.26	28
	ZZ acc-fast	10.53	2.77	11
Error Magnitude	F8 acc	36.40	8.65	32.5
	F8 acc-fast	28.25	6.86	29
	ZZ acc	16.6	8.93	40
	ZZ acc-fast	17.73	8.27	33.5

*SD* Standard deviation, *NDI* Neck disability index, *DHIsf* Dizziness handicap inventory short form, *F8* Figure of 8, *ZZ* Zigzag, *acc* accurate velocity, *acc-fast* accurate and fast velocity, *NA* not applicable

### Intrarater reliability

Intra-rater reliability for both raters was perfect for *time* taken (1.0, SEM < 0.01), almost-perfect for *error frequency* and ranged for F8 between 0.81–0.97, (SEM 0.59–2.50) and for ZZ between 0.95–0.99 (SEM 0.09–0.50). Similar values were seen for *error magnitude* (Table 2).

**Table 2:** Intrarater reliability (*n*= 3)

Rater	Variable	Pattern	ICC Agreement	95% CI	SEM
Rater 1	Time	F8	1	1–1	< 0.01
		ZZ	1	1–1	< 0.01
Rater 2		F8	1	1–1	< 0.01
		ZZ	1	1–1	< 0.01
Rater 1	Error frequency	F8	0.81	0.64–0.99	2.50
		ZZ	0.95	0.78–1	0.50
Rater2		F8	0.97	0.92–0.99	0.59
		ZZ	0.99	0.98–0.99	0.09
Rater 1	Error magnitude	F8	0.83	0.83–0.99	2.92
		ZZ	0.97	0.91–0.99	0.59
Rater 2		F8	0.97	0.92–0.98	0.59
		ZZ	1.0	0.99–1	0.07

*ICC* Intraclass Correlation Coefficients for agreement, *95% CI* 95% Confidence interval, *SEM* Standard error of measurement, *F8* figure of 8, *ZZ* zigzag

### Interrater reliability

Interrater reliability for time for both patterns and velocities was perfect (1.0, SEMs from < 0.01 to 0.05), almost-perfect for *error frequency* with F8 ranging from 0.76 to 0.91, (SEMs 0.47 to 1.74), and ZZ = 0.80 to 0.84, (SEMs 0.48 to 0.78). Similar values were seen for *error magnitude* (Table 3).

**Table 3:** Interrater reliability (*n* = 20)

Variable	Pattern	Velocity	ICC of agreement	95% Confidence interval	SEM
Time	F8	Accurate	1	1–1	0.05
		Accurate& fast	1	0.99–1	0.02
	ZZ	Accurate	1	1–1	< 0.01
		Accurate& fast	1	0.98–1	0.03
Error frequency	F8	Accurate	0.76	0.63–0.86	1.74
		Accurate& fast	0.91	0.86–0.94	0.47
	ZZ	Accurate	0.80	0.60–0.88	0.78
		Accurate& fast	0.84	0.70–0.92	0.48
Error magnitude	F8	Accurate	0.78	0.62–0.86	2.02
		Accurate& fast	0.87	0.77–0.92	1.05
	ZZ	Accurate	0.88	0.68–0.93	0.82
		Accurate& fast	0.94	0.85–0.97	0.69

*ICC* Intraclass Correlation Coefficients for agreement, *SEM* Standard error of measurement, *F8* figure of 8, *ZZ* zigzag

### Feasibility

**Fig. 2:**
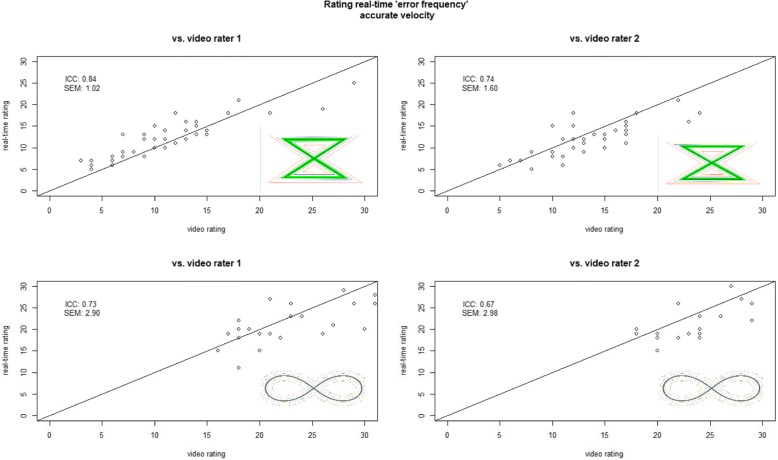
Feasibility of rating error frequency performed by subjects at accurate velocity. ICC = Intraclass Correlation Coefficient, SEM = Standard error measurement

**Fig. 3:**
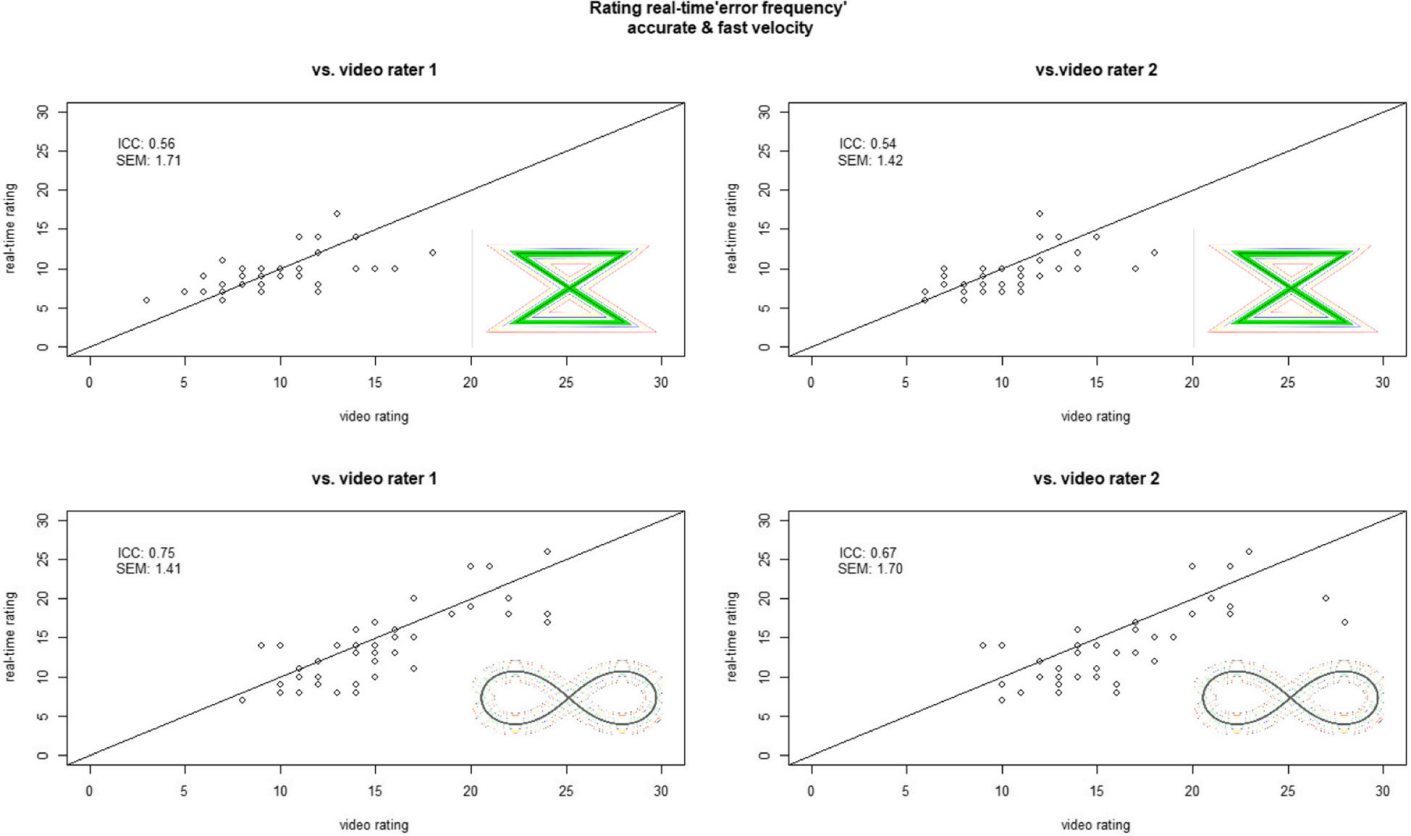
Feasibility of rating error frequency performed by subjects at accurate& fast velocity. ICC = Intraclass Correlation Coefficient, SEM = Standard error measurement

Real-time compared to both video slow motion ratings agreements were almost-perfect for *time* with ICCs between 0.99 to 1.0 (SEMs < 0.01 to 0.05) for both pattern and velocities. For *error frequency* moderate to almost-perfect agreements were shown but overall higher ICCs and lower SEMs were found for ZZ with accurate velocity, while lowest agreement was found for ZZ with accurate & fast velocity and largest SEM values were shown for F8 and accurate velocity. Overall, the real-time ratings of R3 agreed better with the slow motion ratings of R1 than R2 (Table 4, Figs. 2 and 3).

**Table 4:** Feasibility real time rating vs. video rating (*n* = 20)

Comparison	Outcome variable	Pattern	Velocity	ICC agreement (95% CI)	95% CI	SEM
Rater 1 vs. real time	Time	F8	Accurate	1	1–1	< 0.01
			Accurate & Fast	1	1–1	< 0.01
		ZZ	Accurate	1	1–1	< 0.01
			Accurate & Fast	0.99	0.98–1	0.04
	Error Frequency	F8	Accurate	0.73	0.58–0.82	2.90
			Accurate &Fast	0.75	0.61–0.83	1.41
		ZZ	Accurate	0.84	0.72–0.90	1.02
			Accurate &Fast	0.56	0.36–0.70	1.71
Rater 2 vs. real-time	Time	F8	Accurate	1	0.99–1	0.05
			Accurate &Fast	1	1–1	0.02
		ZZ	Accurate	1	1–1	< 0.01
			Accurate &Fast	1	0.99–1	0.02
	Error frequency	F8	Accurate	0.67	0.50–0.78	2.98
			Accurate &Fast	0.67	0.53–0.78	1.70
		ZZ	Accurate	0.74	0.60–0.82	1.60
			Accurate &Fast	0.54	0.39–0.69	1.42

*ICC* Intraclass Correlation Coefficients for agreement, *95% CI* 95% Confidence interval, *SEM* Standard error of measurement, *F8* figure of 8, *ZZ* zigzag

## Discussion

This study demonstrated promising intra and inter-rater reliability and clinical feasibility for assessing the performance of the F8 and ZZ cervical movement sense tests performed by people with neck pain. Overall, the combined results, considering intra and inter rater accuracy and feasibility, suggest that the time taken and frequency of errors during the accurate task, particularly using the ZZ pattern, has the most potential for clinical use.

Our study showed the best **reliability** (both intra- and inter-rater) and feasibility was in rating the *time* subjects needed to perform the tasks. Almost-perfect intra-rater and substantial almost-perfect inter-rater reliability was demonstrated for *error frequency* and *error magnitude*. Tracing the ZZ pattern was slightly more reliable than for the F8 pattern (better ICCs and lower SEMs)*.* Further, *error magnitude* was not feasible for real-time ratings, which may point to *time* and *error frequency* being most useful in the clinical situation.

Encouragingly, similar inter-rater reliability values for *error frequency* (ICC = 0.93) were shown in the Australian study of asymptomatic controls who overall demonstrated less mean errors than the neck pain subjects in the current study [21]. Furthermore, intra-rater reliability shown in our study is comparably high to values reported for rating similar test procedures like joint position error (JPE) measurements [36, 37]. In a study requiring head repositioning after neck rotation or flexion/extension returning to a neutral and target head position, similar ICCs and SEMS to our results were reported (intra: ICC between 0.70–0.83, SEM 1.45–2.45; inter: 0.62–0.84, SEM 1.50–2.23) [36]. Juul et al. [37] reported lower ICCs but better SEMs in examining the reliability of rating JPE returning to a neutral head position from rotation, extension and flexion (intra: ICC 0.48–0.82, SEM 0.19–0.26; inter: ICC 0.50–0.75, SEM 0.20–0.50). Within this context, our almost-perfect intra-rater and substantial to almost-perfect inter-rater reliability of *error frequency* and *magnitude* slow motion video ratings in the current study appear to be excellent results.

The **feasibility** of achieving reliable ratings at real-time in clinic is essential given the complexity and inefficiency of videoing patients and rating them later. The feasibility of error counting during F8 tracing was similar for both velocities; however, the accurate velocity showed larger SEMs, which may relate to the total amount of errors that were more than double for F8 compared to ZZ tracing with accurate velocity, while the time needed to trace each pattern increased equivalently. The F8 pattern central line was narrower and may have related to increased error, while the ZZ accurate task seemed easier for our raters to follow; yet, challenging enough for the patients. Despite better inter-rater reliability, the accurate & fast ZZ tracking appeared, less feasible for assessing in real time with ICCs for error frequency of 0.54 and 0.56 (Table 4), respectively. SEMs of 1.42 and 1.71 (Table 4) in relation to a range of eleven (Table 1) would also support this. Thus considering all of the results, evaluation of *error frequency and time* for the ZZ pattern traced within an accurate velocity appears to be the most promising task for application in clinical practise.

Future directions with respect to test-retest reliability of subjects’ performance and validity of the measures can now be explored [31, 38]. Comparison of our results to those given for asymptomatic controls by Pereira et al. suggest similar results for time to trace each pattern and velocity, but lower error frequency and magnitude values to those found in our neck pain group [21]. The current study revealed nearly twice as many errors on average in neck pain patients for the ZZ pattern, and close to three times the amount of errors during F8 tracing with accurate velocity. This is a promising indication that this simple pattern-tracing assessment of CMS may differentiate between people with and without neck pain. Future case-control comparative studies appear warranted in addition to the test-retest subject reliability studies proposed.

### Limitations of the study

There were limitations to our study that should be considered in interpreting our results. The line thickness for F8 and ZZ were not equal and may have influenced subjects’ performance and reliability. Perhaps accordingly, our neck pain patients demonstrated more errors and needed longer for the F8 (5 mm) than the ZZ pattern (10 mm). In addition, feasibility testing may have been subject to expectation bias in R3 when reconciling disagreement between R1 and R2; however, if applicable, its influence would be low as only 25% of observations disagreed, there was 3–5 weeks between ratings, and R3 was blind to her real-time ratings of those subjects.

Finally, the aim of our study was to determine the intra and inter-rater reliability and feasibility of assessing the patient performing the tasks. A necessary progression will be to compare responses between neck pain and asymptomatic control subjects and examine the reliability of subjects’ repeatable performance, which may influence the responsiveness of the measure and future use of these assessments [20, 39].

## Conclusions

Rating the time taken and number of errors during tasks designed to assess cervical movement sense is reliable (intra and inter tester) and seems feasible for use in clinical practice. Rating of videos in slow motion, for time, error frequency and magnitude, of participants tracing a F8 or ZZ pattern with a head-mounted laser is reliable. Real time rating of *Time* and *error frequency* of an accurately traced ZZ pattern seems most feasible for clinical practise. The results of this study support directions for future research to understand whether these simple movement sense tests allow for meaningful distinction of neck pain, and between sub-groups of this prevalent musculoskeletal condition. A further direction is to determine test validity and within-subject test-retest repeatability.

## Abbreviations

DHI: Dizziness handicap inventory
F8: Figure of eight pattern
JPE: Joint position Error
NDI: Neck disability index
SD: Standard deviation
SEM: Standard error of measurement
WAD: Whiplash associated disorder
ZZ: Zigzag pattern

## References

[1] Hoy D, March L, Woolf A, Blyth F, Brooks P, Smith E, Vos T, Barendregt J, Blore J, Murray C (2014). The global burden of neck pain: estimates from the global burden of disease 2010 study. Ann Rheum Dis.

[2] Vos T, Barber RM, Bell B, Bertozzi-Villa A, Biryukov S, Bolliger I, Charlson F, Davis A, Degenhardt L, Dicker D, et al. Global, regional, and national incidence, prevalence, and years lived with disability for 301 acute and chronic diseases and injuries in 188 countries, 1990–2013: a systematic analysis for the Global Burden of Disease Study 2013. Lancet. 2015;386:743–800. 10.1016/S0140-6736(15)60692-4 PMC4561509 26063472

[3] Childs Maj John D., Fritz Julie M., Piva Sara R., Whitman Julie M. (2004). Proposal of a Classification System for Patients With Neck Pain. Journal of Orthopaedic & Sports Physical Therapy.

[4] Stanton TR, Leake HB, Chalmers KJ, Moseley GL (2016). Evidence of impaired proprioception in chronic, idiopathic neck pain: systematic review and meta-analysis. Phys Ther.

[5] Jull Gwendolen, Sterling Michele, Falla Deborah, Treleaven Julia, O'Leary Shaun (2008). Future Directions. Whiplash, Headache, and Neck Pain.

[6] Kristjansson E, Treleaven J (2009). Sensorimotor function and dizziness in neck pain: implications for assessment and management. J Orthop Sports Phys Ther.

[7] Kulkarni V, Chandy MJ, Babu KS (2001). Quantitative study of muscle spindles in suboccipital muscles of human foetuses. Neurol India.

[8] Liu JX, Thornell LE, Pedrosa-Domellof F (2003). Muscle spindles in the deep muscles of the human neck: a morphological and immunocytochemical study. J Histochem Cytochem.

[9] Treleaven J (2008). Sensorimotor disturbances in neck disorders affecting postural stability, head and eye movement control. Man Ther.

[10] McLain RF (1994). Mechanoreceptor endings in human cervical facet joints. Spine.

[11] Richmond FJ, Bakker DA (1982). Anatomical organization and sensory receptor content of soft tissues surrounding upper cervical vertebrae in the cat. J Neurophysiol.

[12] Roijezon U, Clark NC, Treleaven J (2015). Proprioception in musculoskeletal rehabilitation. Part 1: basic science and principles of assessment and clinical interventions. Man Ther.

[13] Clark NC, Roijezon U, Treleaven J (2015). Proprioception in musculoskeletal rehabilitation. Part 2: clinical assessment and intervention. Man Ther.

[14] Treleaven J, Chen X, Sarig Bahat H (2016). Factors associated with cervical kinematic impairments in patients with neck pain. Man Ther.

[15] Treleaven J (2017). Dizziness, unsteadiness, visual disturbances, and sensorimotor control in traumatic neck pain. J Orthop Sports Phys Ther.

[16] Michiels S, De Hertogh W, Truijen S, November D, Wuyts F, Van de Heyning P (2013). The assessment of cervical sensory motor control: a systematic review focusing on measuring methods and their clinimetric characteristics. Gait Posture.

[17] Sarig Bahat H, Weiss PL, Laufer Y (2010). The effect of neck pain on cervical kinematics, as assessed in a virtual environment. Arch Phys Med Rehabil.

[18] Sarig Bahat H, Chen X, Reznik D, Kodesh E, Treleaven J (2015). Interactive cervical motion kinematics: sensitivity, specificity and clinically significant values for identifying kinematic impairments in patients with chronic neck pain. Man Ther.

[19] Woodhouse A, Stavdahl O, Vasseljen O (2010). Irregular head movement patterns in whiplash patients during a trajectory task. Exp Brain Res.

[20] Kristjansson E, Oddsdottir GL (2010). "The Fly": a new clinical assessment and treatment method for deficits of movement control in the cervical spine: reliability and validity. Spine.

[21] Pereira MJ, Beaudin C, Grewal G, Wong V, Treleaven J. Cervical movement sense: normative data for a clinical tool. In: Australian physiotherapy association conference: "New moves" Data provided by the first author as presented at the Conference. Melbourne: Australian physiotherapy association; 2013.

[22] Meisingset I, Woodhouse A, Stensdotter A-K, Stavdahl Ø, Lorås H, Gismervik S, Andresen H, Austreim K, Vasseljen O (2015). Evidence for a general stiffening motor control pattern in neck pain: a cross sectional study. BMC Musculoskelet Disord.

[23] MacDermid JC, Walton DM, Avery S, Blanchard A, Etruw E, McAlpine C, Goldsmith CH (2009). Measurement properties of the neck disability index: a systematic review. J Orthop Sports Phys Ther.

[24] Vernon H (2008). The neck disability index: state-of-the-art, 1991-2008. J Manip Physiol Ther.

[25] Swanenburg J, Humphreys K, Langenfeld A, Brunner F, Wirth B. Validity and reliability of a German version of the neck disability index (NDI-G). Man Ther. 2013. 10.1016/j.math.2013.07.004 23920153

[26] Spitzer WO, Skovron ML, Salmi LR, Cassidy JD, Duranceau J, Suissa S, Zeiss E (1995). Scientific monograph of the Quebec task force on whiplash-associated disorders: redefining "whiplash" and its management. Spine (Phila Pa 1976).

[27] Carlsson AM (1983). Assessment of chronic pain. I. Aspects of the reliability and validity of the visual analogue scale. Pain.

[28] Kurre A, van Gool CJ, Bastiaenen CH, Gloor-Juzi T, Straumann D, de Bruin ED (2009). Translation, cross-cultural adaptation and reliability of the german version of the dizziness handicap inventory. Otol Neurotol.

[29] Tesio L, Alpini D, Cesarani A, Perucca L (1999). Short form of the dizziness handicap inventory: construction and validation through Rasch analysis. Amer J Phys Med Rehabil.

[30] de Vet HCW, Terwee CB, Knol DL, Bouter LM (2006). When to use agreement versus reliability measures. J Clin Epidemiol.

[31] de Vet HCW, Terwee CB, Mokkink LB, Knol DL (2011). Measurement in medicine: a practical guide.

[32] Landis JR, Koch GG (1977). The measurement of observer agreement for categorical data. Biometrics.

[33] R-Development-Core-Team. R: a language and environment for statistical computing. Vienna: R Foundation for Statistical Computing; 2008.

[34] Falissard B. psy: Various procedures used in psychometry. Vienna: R Foundation for Statistical Computing; 2012.

[35] Canti A, Ripley B. Boot: bootstrap R (S-plus) functions. Vienna: R Foundation for Statistical Computing; 2017.

[36] Alahmari K, Reddy RS, Silvian P, Ahmad I, Nagaraj V, Mahtab M (2017). Intra- and inter-rater reliability of neutral head position and target head position tests in patients with and without neck pain. Braz J Phys Ther.

[37] Juul T, Langberg H, Enoch F, Sogaard K (2013). The intra- and inter-rater reliability of five clinical muscle performance tests in patients with and without neck pain. BMC Musculoskelet Disord.

[38] Streiner DL, Norman GR. Health measurement Scales. 4th ed. Oxford: Oxford University Press; 2008.

[39] Pinsault N, Fleury A, Virone G, Bouvier B, Vaillant J, Vuillerme N (2008). Test-retest reliability of cervicocephalic relocation test to neutral head position. Physiother Theory Pract.

